# Genetic Analysis of* LRRK2* R1628P in Parkinson's Disease in Asian Populations

**DOI:** 10.1155/2017/8093124

**Published:** 2017-10-25

**Authors:** Yuan Zhang, Qiying Sun, Minhan Yi, Xun Zhou, Jifeng Guo, Qian Xu, Beisha Tang, Xinxiang Yan

**Affiliations:** ^1^Department of Neurology, Xiangya Hospital, Central South University, Changsha, Hunan 410008, China; ^2^Department of Geriatrics, Xiangya Hospital, Central South University, Changsha, Hunan 410008, China; ^3^National Clinical Research Center for Geriatric Diseases, Changsha, Hunan 410078, China; ^4^Key Laboratory of Hunan Province in Neurodegenerative Disorders, Central South University, Changsha, Hunan 410008, China; ^5^State Key Laboratory of Medical Genetics, Changsha, Hunan 410078, China; ^6^Institute of Information Security and Big Data, Central South University, Changsha, Hunan 410083, China

## Abstract

Although the etiology of Parkinson's disease (PD) remains unclear, there is increasing evidence of genetic factors contributing to the onset of PD. Various mutations and risk variants of the gene* LRRK2* have been reported, but the association between* LRRK2* R1628P and PD is still inconsistent. Thus, we conducted a meta-analysis to determine the potential relationship between R1628P and PD. Our study sample was an aggregate of 17 publications, which in total consisted of 9,275 PD patients and 8,114 controls. All of these articles are of high quality according to NOS, and there was no obvious reporting bias or heterogeneity. In a general Asian population, the pooled OR of the risk genotype contrasts was 1.83 (95% CI: 1.57, 2.13). When stratified by ethnicity, the pooled ORs were 1.84 (95% CI: 1.56, 2.18) in a Chinese population and 1.79 (95% CI: 1.27, 2.52) in a non-Chinese population. Our study suggests that* LRRK2* R1628P appears to be a risk factor for PD in Asian populations, both Chinese and non-Chinese.

## 1. Introduction

Parkinson's disease (PD) is currently the second most common progressive neurodegenerative disorder in the world. Although current science has not been able to discern the exact causes of PD, there is an increasing evidence that genetic factors contribute to the etiology of PD [1].

Certain mutations and risk variants of the gene* leucine-rich repeat kinase 2 *(*LRRK2*; PARK8) are very common in the PD-afflicted population, accounting for approximately 5–15% of familial and sporadic PD [2]. Besides, the prevalence differs among different populations [3, 4]. For instance, the reported proportion of G2019S carriers [5–7] in Ashkenazi Jews and North African Arabs is more than 30%, whereas it is nearly nonexistent in Asians. Likewise, in Asian populations, the mostly common variants are G2385R and R1628P [8, 9]. Between these two variants, it is the association between R1628P and PD; however, that is inconclusive. Other studies [10–13] have found that R1628P is a contributing factor to PD in mainland China, Taiwan, Malaysia, and Thailand. Conversely, a large multicenter study [14] and several other investigators [15–17] did not identify any associated risk of R1628P in either East Asian, Caucasian, or Arab-Berber cohorts.

In order to evaluate the association between R1628P and PD, we used meta-analytic methods to assess the possible role of R1628P in PD in a worldwide context.

## 2. Materials and Methods

### 2.1. Search Strategy

We searched electronic databases including Embase, PubMed, Cochrane Library, Web of knowledge, Wangfang database, and CNKI. We employed this search method until February 1st, 2017, using a combination of the following keywords,* LRRK2*, R1628P, PD, and Parkinso^*∗*^, in both English and Chinese. Reference lists and personal communications of authors were also referred as sources to include articles cited elsewhere. All searched publications were imported to endnote for further management. Only one copy of the same papers, which were included in different databases, was kept to avoid duplication.

### 2.2. Selection Criteria

The following criteria were used to discern the eligibility of a study: (1) the article had to be a case-control study, with the exception of reviews, case reports, editorials, or functional research; (2) all PD patients were diagnosed according to UKBB or other accepted criteria with the exception that positive family history was not a part of the exclusion criteria; (3) all controls were free of symptoms suggestive of PD or other neurological disorders; (4) results of R1628P sequencing were reported both in patients and in controls. We excluded the following studies: (1) duplicate publications and articles which used an overlapping sample and (2) lack of original and sufficient data to calculate the OR.

### 2.3. Data Extraction and Quality Assessment

To select studies for further assessment, two authors independently scanned the abstracts, titles, or both sections of each retrieved record. All potentially relevant articles were investigated in full. For studies satisfying the aforesaid criteria, two authors independently extracted the following data: year of publication, first author's surname, ethnicity of participants, participant's country of origin, number of R1628P carriers, total number of PD patients and healthy controls, and finally the age and gender ratio for both PD patients and controls. The qualities of the included studies were evaluated by the Newcastle–Ottawa Scale (NOS) [18], of which the genotyping method was an essential part in quality rating process.

### 2.4. Statistical Analysis

In order to assess the strength of association between* LRRK2* R1628P and PD, the outcome was expressed as odds ratio (OR) along with a corresponding 95% confidence interval (CI). Heterogeneity across individual studies was identified using a standard *Q* test with a significance level of *α* = 0.1 and *I*^2^. If heterogeneity did not exist (*Q* > 0.10) or the severity of heterogeneity was accepted (*I*^2^ ≤ 50%), then the fixed effect model was adopted to calculate the pooled OR value. Otherwise, the random effect model was used. Funnel-plot analysis was used to assess the reporting bias. All analyses were carried out by using the Review Manager software package v.5.3 (The Cochrane Collaboration, Oxford, England). In addition, subgroup analysis was performed according to ethnicity for all studies. In order to assess the stability of the results, sensitivity analysis was performed by removing each individual study from the combined total and then reanalyzing the remainder.

## 3. Results

### 3.1. Literature Selection and Study Characteristics

**Figure 1:**
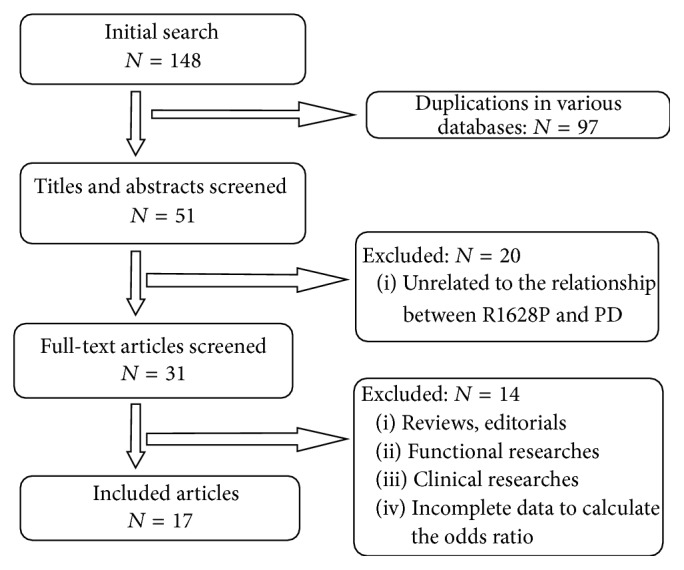
Flow chart of included publications.

During the initial selection stage, we extracted 148 potentially relevant articles from databases stated above. First, we excluded 97 due to duplication in different databases. Then, 20 publications were excluded for irrelevance to both PD and R1628P, which was determined after screening the titles and abstracts. 14 papers were not included for lack of sequencing results of R1628P both in PD patients and in controls, or the data was not enough to calculate the OR. This process culminated to a total of 17 eligible studies included in qualitative analysis and 16 studies included in the further quantitative analysis. The flow chart illustrating the selection procedure is presented in Figure 1. The main characteristics of the included studies are summarized in Table 1. The NOS scores of the included publications were rated from 7 to 9 stars, which attested to their high quality.

### 3.2. Data Synthesis

**Figure 2:**
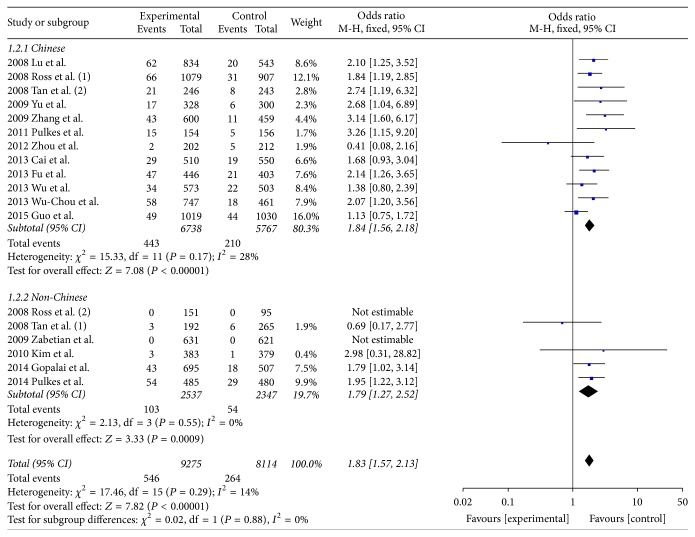
Forest plot of R1628P in PD.

**Figure 3:**
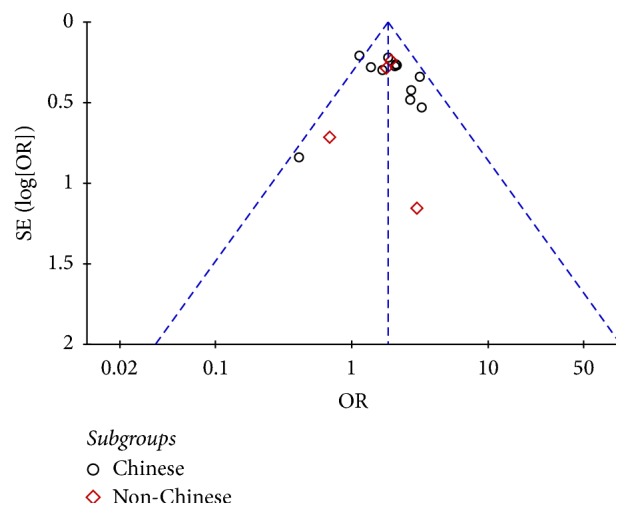
Funnel plot of R1628P in PD.

All of the 17 included studies pertained to Asian populations. The pooled OR for R1628P in Asian was 1.83 (95% CI: 1.57, 2.13) (Figure 2). The funnel plot was symmetry (Figure 3). In a secondary analytic step, we divided all participants into Chinese and non-Chinese groups. Notably, among these chosen databases, the publication by Ross et al. [19] contained both Chinese and non-Chinese populations, which were analyzed individually. Within the Chinese population, there were 6738 cases of PD and 5767 controls. The pooled OR of the Chinese cohort demonstrates a significantly associated risk of R1628P, with the value of 1.84 (95% CI: 1.56, 2.18) (Figure 2). Among the non-Chinese subgroup, there were 2537 PD subjects and 2347 controls. Since no carriers were screened in Japanese [19–24], we conducted the meta-analysis using the other four publications. These publications were studied in Malaysian, Indian, Korean, and Thailand populations. While only the Thailand population demonstrated a significant, independent association, the pooled OR of R1628P in the combined non-Chinese group was 1.79 (95% CI: 1.27, 2.52) (Figure 2). During the sensitivity analysis, there were no significant changes to the outcomes.

## 4. Discussion

In the meta-analysis, a combination of 9275 PD patients and 8114 controls was studied. All included publications were of high quality according to NOS and the results were stable during sensitive analysis. Furthermore, there was no publication reporting bias, as shown by the symmetric funnel plot. It is believed that the final results are credible. The included studies were conducted in mainland China, Taiwan, Singapore, Japan, Malaysia, India, Korea, and Thailand. We found that R1628P carriers were reported as simply being a part of the general Asian population and then subsequently divided them into Chinese and non-Chinese for further stratified analysis. Even though a variety of Asian countries were included, the heterogeneity of the sample was accepted with *I*^2^ less than 50%. Therefore, based on the evidence, we found that* LRRK2 *R1628P was a risk factor for PD in Asian, `both Chinese and non-Chinese, populations.

Although the cause of PD is unclear, it is accepted that its etiology is the interaction of genetic and environmental factors, along with genetic insights which provide a way to explore the molecular causes of PD. As a result, more and more attention has been focused on the relationship between genetics and PD.* LRRK2*, one of the genes related to autosomal dominant form of PD, belongs to a member of the ROCO protein family [29] and has five major functional domains [30]: a leucine-rich repeat (LRR); a Roc domain (Ras in complex proteins); a COR domain (C-terminal of Roc); a TyrKc domain (tyrosine kinase catalytic), and WD40 domain. The variations of* LRRK2* account for approximately 5–15% of familial and sporadic PD [2]. Additionally, further studies show that various variants, such as G2019S and G2385R, have genetic correlations and heterogeneity. This means that different variations, including mutations and polymorphism, may play a diverse role depending on the given population.

R1628P, a substitution of a polar arginine (R) with a neutral nonproline (P), is located in the COR domain of LRRK2. This kind of substitution could change the secondary structure of LRRK2 and influence the interaction between the other domains of LRRK2 or different external proteins, which ultimately influences the kinase activity [31, 32]. In fact, a study in our team once reported that R1628P was not associated with PD risk in mainland China [28] (PD = 1019, *N* = 1030), which was consistent with [11, 27] or contrary to [12, 13, 22] other similar studies conducted. We suppose that a larger number of participants would be helpful for observing the actual results. To draw a comprehensive understanding, we performed a meta-analysis to evaluate the contribution of* LRRK2 *R1628P to PD worldwide. And we found* LRRK2* R1628P was a risk factor in Asian ethnicities, which may help for further pathogenetic exploring of* LRKK2*, especially in the Asian population. Additionally, Shu's study identified that R1628P could be an example that explains the genetic-environmental interaction. Instead of upregulation of the kinase activity of LRRK2 directly, it was found that the status of R1628P in* LRRK2* provides a potential “two-hit” target: firstly environmentally toxic induced Cdk5 activation and then Cdk5 phosphorylating the adjacent amino residue S1627 of the R1628P mutation; thus LRRK2 kinase was activated which leads to the death of neurons.

The International Parkinson and Movement Disorder Society (MDS) Task Force proposed that PD should be divided into three stages [33]: preclinical PD, prodromal PD, and clinical PD. The stage of prodromal PD [34] refers to the presentation of symptoms or signs of PD neurodegeneration with absence of classic motor parkinsonism. Genetics findings, combined with age, environmental risk factors, and other diagnostic marker tests, play a vital role in the diagnosis of prodromal PD [34, 35]. We found* LRRK2 *R1628P was a risk factor in Asian populations, both Chinese and non-Chinese. This may help for further exploring etiology and biomarker of PD, especially in Asian population.

A few limitations of this study should be acknowledged: firstly, as all data were extracted from previously published papers, we do not know the effects of the unpublished articles. Secondly, some articles [14, 36, 37], which otherwise met the eligibility criteria, were excluded for insufficient data. Thirdly, other confounding factors, like age, gender, and age at onset, may be affecting the outcomes and prevalence of PD.

In conclusion, our research showed a significant association between R1628P and the susceptibility to PD in the Asian population, both Chinese and non-Chinese. Further investigation of high quality, large cohort studies and function researches is needed to elaborate this relationship.

## Figures and Tables

**Table 1:** Attributes of all included studies.

Publications	Ethnicity	Region	Total number of cases/controls	R1628P carriers of case/control	Age of case/control	Male ratio of case/control	NOS
2008 Lu et al. [17]	Chinese	Taiwan	843/543	62/20	65.7 ± 11.8/51.9 ± 18.4	58.9/41.3	8
2008 Ross et al. [19]	Chinese	Taiwan and Singapore	1079/907	66/31	NA	NA	8
2008 Ross et al. [19]	Non-Chinese	Japan	151/95	0/0	NA	NA	8
2008 Tan et al. (2) [20]	Chinese	Singapore	246/243	21/8	66 ± 12/62 ± 10	56.0/54.0	8
2008 Tan et al. (1) [21]	Non-Chinese	Malaysia and Indian	192/265	3/6	60.3 ± 6.4/64.5 ± 3.7	NA	9
2009 Zhang et al. [22]	Chinese	China	600/459	43/11	NA/53.4 ± 13.4	57.0/56.0	7
2009 Yu et al. [23]	Chinese	China	328/300	17/6	NA/58.2 ± 10.3	54.3/57.7	9
2009 Zabetian et al. [24]	Non-Chinese	Japan	631/320	0/0	NA	NA	9
2010 Kim et al. [15]	Non-Chinese	Korea	383/379	3/1	NA	NA	8
2011 Pulkes et al. [25]	Thai/Chinese	Thai	154/156	15/5	NA	56.5/NA	8
2012 Zhou et al. [16]	Chinese	China	202/212	2/5	62.68 ± 10.69/62.87 ± 10.44	52.5/59.0	8
2013 Fu et al. [13]	Chinese	China	446/403	47/21	60.82 ± 11.20/59.21 ± 9.3	59.0/62.0	9
2013 Wu-Chou et al. [12]	Chinese	Taiwan	747/461	58/18	NA	NA	8
2013 Cai et al. [26]	Chinese	China	510/550	29/19	58.4 ± 11.0/59.34 ± 11.3	59.4/58.2	9
2013 Wu et al. [27]	Chinese	Taiwan	573/503	34/22	62.1 ± 11.5/59.4 ± 12.9	55.3/50.7	8
2014 Pulkes et al. [11]	Non-Chinese	Thai	485/480	54/29	65.0 ± 12.0/71.0 ± 7.0	46.0/61.0	9
2014 Gopalai et al. [10]	Non-Chinese	Malaysia	695/507	43/18	57.4 ± 11.8/59.3 ± 9.4	60.0/51.0	8
2015 Guo et al. [28]	Chinese	China	1019/1030	49/44	57.9 ± 12.3/57.2 ± 16.4	58.5/48.0	9

NOS: Newcastle–Ottawa Scale. NA: not available.
